# miR-23b as a potential tumor suppressor and its regulation by DNA methylation in cervical cancer

**DOI:** 10.1186/s13027-015-0037-6

**Published:** 2015-11-30

**Authors:** Gabriela Elizabeth Campos-Viguri, Hilda Jiménez-Wences, Oscar Peralta-Zaragoza, Gricenda Torres-Altamirano, Diana Guillermina Soto-Flores, Daniel Hernández-Sotelo, Luz Del Carmen Alarcón-Romero, Marco Antonio Jiménez-López, Berenice Illades-Aguiar, Gloria Fernández-Tilapa

**Affiliations:** Laboratorio de Investigación Clínica, Unidad Académica de Ciencias Químico Biológicas, Universidad Autónoma de Guerrero, Av. Lázaro Cárdenas S/N, Ciudad Universitaria, Colonia La Haciendita, C.P. 39089 Chilpancingo, Guerrero México; Instituto Nacional de Salud Pública, Avenida Universidad No. 655, Colonia, Santa María Ahuacatitlán, Cuernavaca, Morelos C.P. 62100 México; Laboratorio de Virología y Epigenética del Cáncer, Unidad Académica de Ciencias Químico Biológicas, Universidad Autónoma de Guerrero, Av. Lázaro Cárdenas S/N, Ciudad Universitaria, Colonia La Haciendita, C.P. 39089 Chilpancingo, Guerrero México; Laboratorio de Investigación en Citopatología e Histoquímica, Unidad Académica de Ciencias Químico Biológicas, Universidad Autónoma de Guerrero, Av. Lázaro Cárdenas S/N, Ciudad Universitaria, Colonia La Haciendita, C.P. 39089 Chilpancingo, Guerrero México; Instituto Estatal de Cancerología “Dr. Arturo Beltrán Ortega”, Av. Adolfo Ruiz Cortines No. 128-A, Colonia Alta Progreso, Acapulco de Juárez, Guerrero C.P. 39570 México; Laboratorio de Biomedicina Molecular, Unidad Académica de Ciencias Químico Biológicas, Universidad Autónoma de Guerrero, Av. Lázaro Cárdenas S/N, Ciudad Universitaria, Colonia La Haciendita, Chilpancingo, Guerrero C.P. 39089 México

**Keywords:** expression, miR-23b, DNA methylation, cervical cancer, HPV16, *upa*, *c-met*, *zeb1*

## Abstract

**Background:**

The aberrant expression of miR-23b is involved in the development and progression of cancer. The aim of this study was to evaluate the potential role of methylation in the silencing of miR-23b in cervical cancer cell lines and to determine its expression in stages of malignant progression and in cervical cancer tissues HPV16-positive.

**Methods:**

The methylation of the miR-23b promoter was determined in HeLa, SiHa, CaSki and C33A cells using a Human Cancer miRNA EpiTectMethyl II Signature PCR Array®. The cells were treated with 5-Aza-2′-deoxycytidine, and the expression of miR-23b, *uPa, c-Met* and *Zeb1* was determined by qRT-PCR. miR-92a and GAPDH were used as controls. The expression of miR-23b was determined in cervical scrapes and biopsies of women without squamous intraepithelial lesions, with precursor lesions and with cervical cancer, all were HPV16-positive. The Fisher exact and Mann–Whitney tests were used to compare the differences of the expression of miR-23b, *uPa, c-Met* and *Zeb1* among cell groups, and the difference among patients, respectively. The association between the expression of miR-23b and cervical cancer was determined by logistic regression with a confidence level of 95 %. A value of p < 0.05 was considered statistically significant.

**Results:**

In C33A, HeLa and CaSki cells, methylation was associated with decreased expression of miR-23b. After treatment with 5-Aza-CdR, the expression of miR-23b increased in all cell lines and the expression of *c-Met* decreased in HeLa cells, while *uPa* and *Zeb1* decreased in C33A and CaSki cells. In SiHa cells the expression of *uPa*, *c-Met* and *Zeb1* increased. The expression of miR-23b decreased in relation to the increase in the severity of the lesion and was significantly lower in cervical cancer. In women with premalignant lesions HPV16-positive, decreased levels of miR-23b increased the risk of cervical cancer (OR = 36, 95 % CI = 6.7-192.6, p < 0.05).

**Conclusions:**

The results suggest that the expression of miR-23b is regulated by the methylation of its promoter and is possible that this microRNA influence the expression of *uPa, c-Met* and *Zeb1* in cervical cancer cells lines. In women with premalignant lesions and cervical cancer infected with HPV16, the expression level of miR-23b agree with a tumor suppressor gene.

## Background

The high-risk human papillomavirus (HR-HPV) causes cervical cancer [1], and this infection is also associated with precancerous lesions of the cervix [2, 3]. In addition, other molecular events such as genetic and epigenetic abnormalities also contribute to the transformation and immortalization of epithelial cells infected with HR-HPV [4].

The miRNAs can regulate 60 to 90 % of the protein-encoding genes and a single miRNA can regulate, directly or indirectly, the expression of hundreds of target mRNAs [5–7]. The aberrant expression of miRNAs has been associated with the maintenance of the undifferentiated state of cancer cells [8].

miRNA biogenesis is highly regulated by multiple processes. Approximately 40 % of human miRNAs are organized in conserved clusters, with distances of at least 5000 bp between them [9], and these miRNAs are co-transcribed as discrete polycistronic pri-miRNAs [6, 10]. Although the miR-23b gene is encoded in the human chromosome 9q22.32 in a cluster that includes miR-24-1 and miR-27b, the mature sequences of each miRNA are differentially expressed [11, 12].

The altered expression of miR-23b has been found to be associated with many types of cancer. In breast cancer, the overexpression of miR-23b is correlated with cell proliferation and metastasis, and is thus recognized as an oncogene [13]. In contrast, the expression of miR-23b in breast cancer increases the formation of focal adhesions and cell-cell junctions, thereby indicating a metastatic suppressor role for this microRNA [14]. Furthermore, the expression of miR-23b has been found to be decreased in castration-resistant prostate cancer tissue, while its overexpression suppresses migration and invasion. Thus, miR-23b is also recognized as a metastatic suppressor in this type of cancer [6, 7, 9].

The expression of miR-23b is decreased in HR-HPV positive cervical cancer tissue and cell lines [15], and it is associated with the overexpression of the urokinase-type plasminogen activator (uPA), which is target gene of this miRNA [16]. uPA has been directly associated with migration and invasion in cervical cancer [17]. Although it is proposed that the E6 oncoprotein of HR-HPV regulates the expression of miR-23b in cervical cancer, this mechanism has not been fully elucidated.

A significant number of miRNAs are embedded in CpG islands and that they are targets of epigenetic regulation [8, 17–19]. In human neoplasia, hypermethylation of CpG sites is associated with transcriptional silencing of tumor suppressor genes and genes that encode miRNAs [20]. There is evidence that miR-23b is subjected to epigenetic silencing in glioblastoma and prostate cancer [7, 8]. Interestingly, it has been suggested that, HPV16 E6 increases the levels of DNA methyltransferase 1 (DNMT1) by degradation of p53 in cervical cancer, causing hypermethylation of miRNA genes, among others [21].

Is proposed that in cervical cancer the inhibition of p53 expression by HR-HPV E6 contributes to the decreased expression of miR-23b. The presence of CpG sites in the promoter sequence of miR-23b allows the regulation of this miRNA by methylation. It is known that epigenetic silencing of miRNAs is associated with processes of invasion and metastasis. Identifying the mechanism by which the expression of miR-23b is regulated in cervical cancer will provide useful information to increase the understanding of this pathology and to create therapies targeting specific epigenetic modifications.

The objective of this research was to evaluate the potential role of methylation in the silencing of miR-23b in cervical cancer cell lines and to determine the pattern of expression of this microRNA in cervical tissues of patients without squamous intraepithelial lesion (Non-SIL), with precursor lesions and HPV16 cervical cancer. In this study, we found decreased expression of miR-23b in cervical tissue with premalignant lesions in cervical cancer and in cervical cancer cell lines. Treatment with 5′-aza-2-deoxycytidine restored the expression of miR-23b in C33A, HeLa and CaSki cells.

## Results

### Model of the elements regulating the expression of miR-23b

**Fig. 1:**
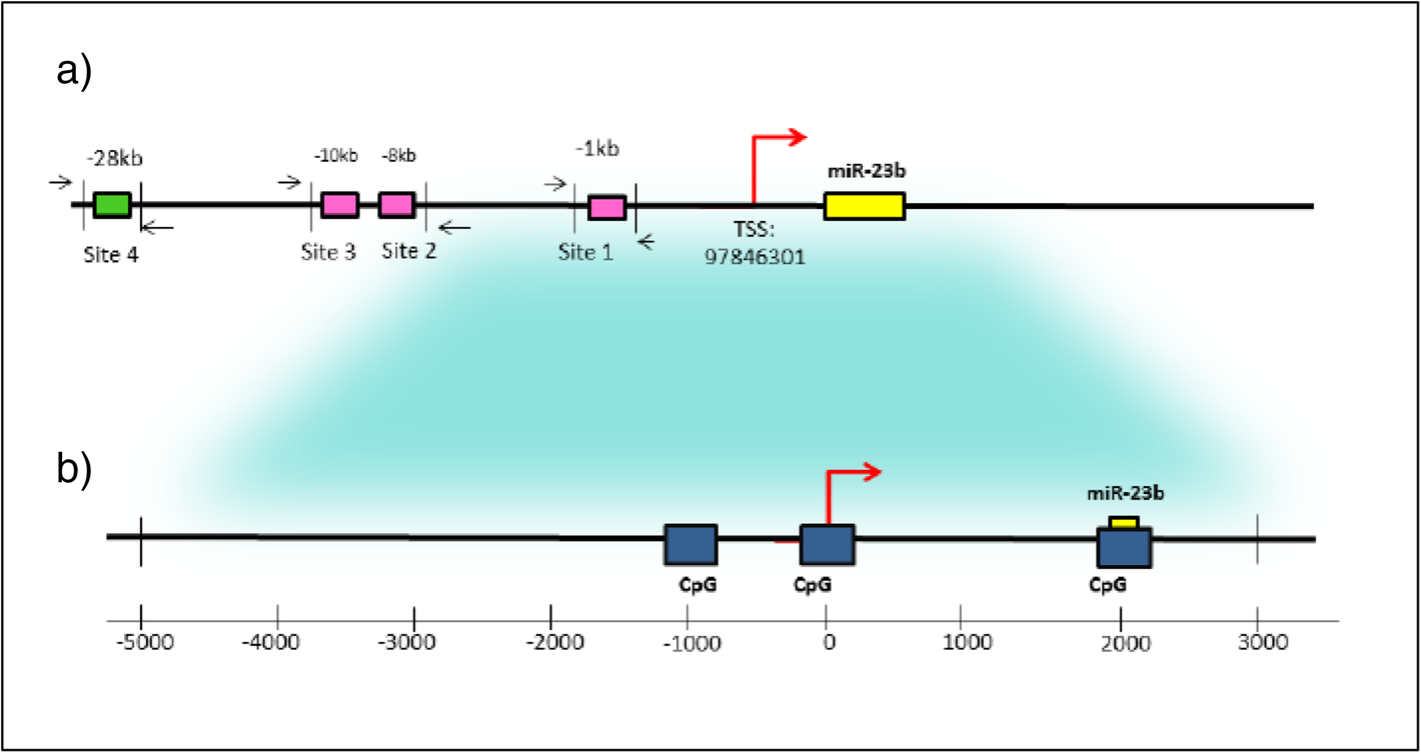
Regulatory elements for the expression of miR-23b. **a** The diagram shows putative p53-binding sites in the miR-23b promoter. The yellow box represents the location of miR-23b precursor, and the green box symbolizes a binding site for p53 [22]. The pink boxes indicate putative binding sites for p53 [22, 23]. **b** CpG islands in the promoter region of miR-23b (blue boxes). The region containing CpG islands was amplified in this study to determine the methylation status of miR-23b promoter

An extensive review of the literature was conducted for the selection of miR-23b. We constructed a model of the regulatory region of the miR-23b gene based on the information found. Some elements that may contribute to the regulation of expression of this microRNA are highlighted in this model as follows:

- The promoter region of miR-23b gene is located in CpG dinucleotide-rich area [7, 8].
- In the first kilobase, there are two CpG islands with high CpG density upstream of the transcription start site (TSS) of miR-23b [7].
- The regulatory sequence of miR-23b, contains four putative sites representing response elements for p53 at 1, 8, 10 and 28 kb upstream of the transcription start site of miR-23b [5, 22, 23] (Fig. 1).

### The expression level of miR-23b is decreased in cervical cancer cell lines due to the methylation of its promoter region

**Fig. 2:**
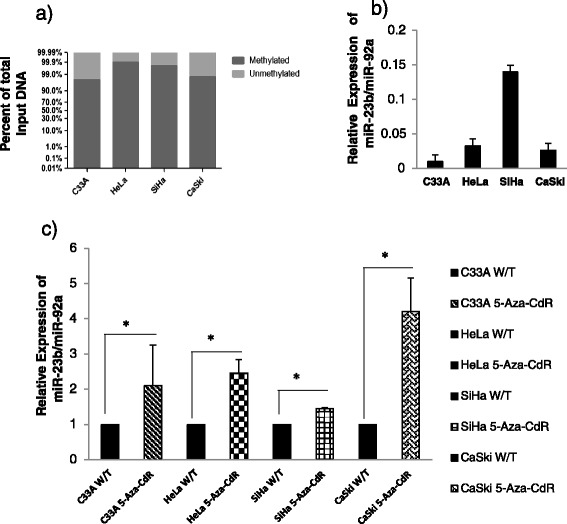
miR-23b is deregulated by methylation of its promoter region in cervical cancer cell lines. **a** The analysis of the methylation status revealed the almost 100 % of total input DNA had methylated miR-23b gene promoter in C33A, HeLa, SiHa and CaSki cells untreated with 5′-Aza-CdR. **b** The relative expression levels of miR-23b were decreased in C33A, HeLa and CaSki cells compared to the levels observed in SiHa cells. **c** Treatment with 10 μM 5′-Aza-CdR significantly increased the expression of miR-23b in the four cell lines (weft bars) compared to untreated cells (black bars). The increased relative expression of miR-23b was ≥50 % over the initial expression (W/T) in C33A, HeLa and CaSki but not in SiHa cells. *Statistically significant difference (p < 0.05); W/T: Untreated cells

Bioinformatics analysis and published data allowed us to identify miR-23b as a candidate for regulation by methylation of its promoter region in cervical cancer. The methylation status of the promoter region of miR-23b in HeLa, SiHa, CaSki and C33A cell lines was evaluated by qRT-PCR. Methylation of the miR-23b promoter was close to 100 % in the four cell lines (Fig. 2a).

To investigate if the methylation status corresponds to the expression level of miR-23b, we determined the relative expression of miR-23b in HeLa, SiHa, CaSki and C33A cells. In C33A, HeLa and CaSki cells, the low expression of miR-23b was negatively related with methylation of the promoter of this miRNA. In contrast, SiHa cells showed a higher level of miR-23b expression than C33A, HeLa and CaSki cells (Fig. 2b).

Furthermore, to confirm that methylation is a mechanism that affects the expression of miR-23b in HeLa, SiHa, CaSki and C33A cells, the cells were exposed to 5′-Aza-CdR, a compound that inhibits DNA methylation. Compared with untreated cells, the expression level of miR-23b was higher in all cell lines after treatment with 5′-Aza-CdR. The increase in the expression of miR-23b was statistically significant in C33A, HeLa, SiHa and CaSki cells (p < 0.05), (Fig. 2c).

### Effect of exposure of cervical cancer cell lines to 5′-Aza-CdR on potential molecular targets of miR-23b

**Fig. 3:**
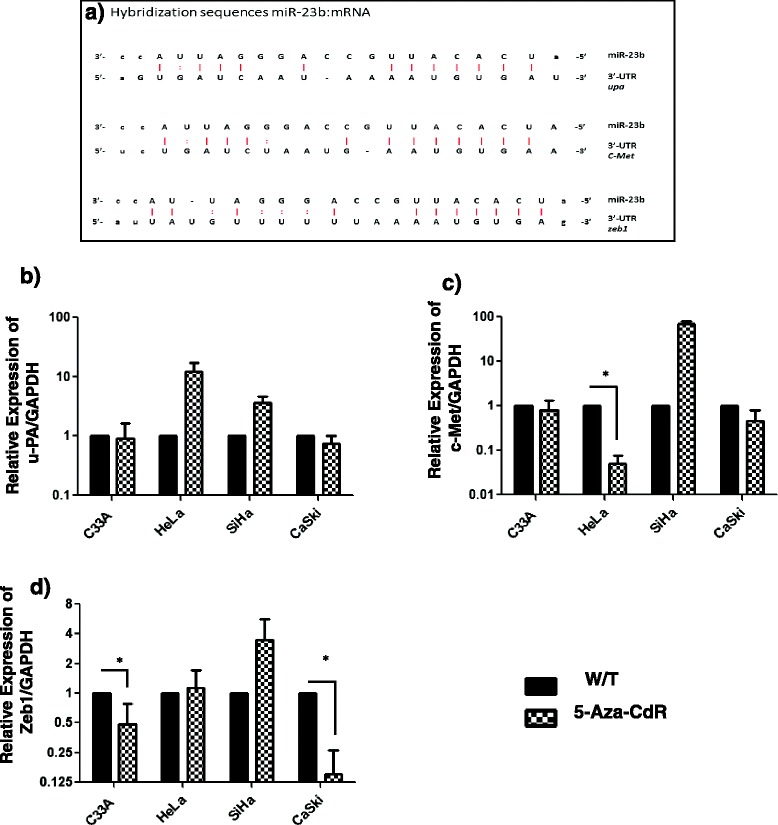
Differential mRNA expression of uPa, c-Met and Zeb1 in cervical cancer cell lines treated with 5′-Aza-CdR. **a** Hybridization sequences between miR-23b and its potential target mRNAs. **b** After treatment with 5′-Aza-CdR, the mRNA expression level of uPa decreased in C33A and CaSki cells. **c** The expression of c-Met decreased in C33A, CaSki and HeLa cells after exposure to 5′-Aza-CdR and the changes in HeLa cells were significant. **d** Treatment with 5′- Aza-CdR significantly decreased Zeb1 expression levels in C33A and CaSki cells. The mRNA expression levels of GAPDH were used to normalize the data. *Statistically significant difference (p < 0.05); W/T: Untreated cells

The information available in the literature and from the *miRanda* and *TargedScan* databases indicates that *uPa, c-Met* and *Zeb1* mRNAs contain miR-23b-binding sequences (Fig. 3a).

To verify that the observed changes in the expression level of miR-23b influence the expression of *uPa*, *c-Met* and *Zeb1,* which are likely targets of this microRNA, the relative mRNA expression of the three genes was determined in HeLa, SiHa, CaSki and C33A cells before and after exposure to 5′-Aza-CdR.

The hypomethylating treatment with 5′-Aza-CdR decreased the mRNA expression of uPa in C33A and CaSki cells but not in HeLa and SiHa cells. Furthermore, the expression of c-Met was significantly reduced only in HeLa cells after treatment. The mRNA expression of Zeb1 decreased only in C33A and CaSki cells, showing significant changes in both cell lines treated with 5′-Aza-CdR. In SiHa cells, the expression levels of uPa, c-Met and Zeb1 increased significantly after exposure to 5-Aza-CdR. The expression of these genes in SiHa cells may be dysregulated by epigenetic factors among other modulation mechanisms, (Fig. 3b, c and d).

### Expression of miR-23b in scrapes from non-SILs, with premalignant lesions and cervical cancer

To investigate whether the expression level of miR-23b was similar in cell lines and in samples from the cervix, we analyzed cervical scrapes from non-SILs (n = 18) and tissues from patients diagnosed with LSILs (n = 19), HSILs (n = 7) or cervical cancer (n = 28), all patients were infected with HPV16.

**Fig. 4:**
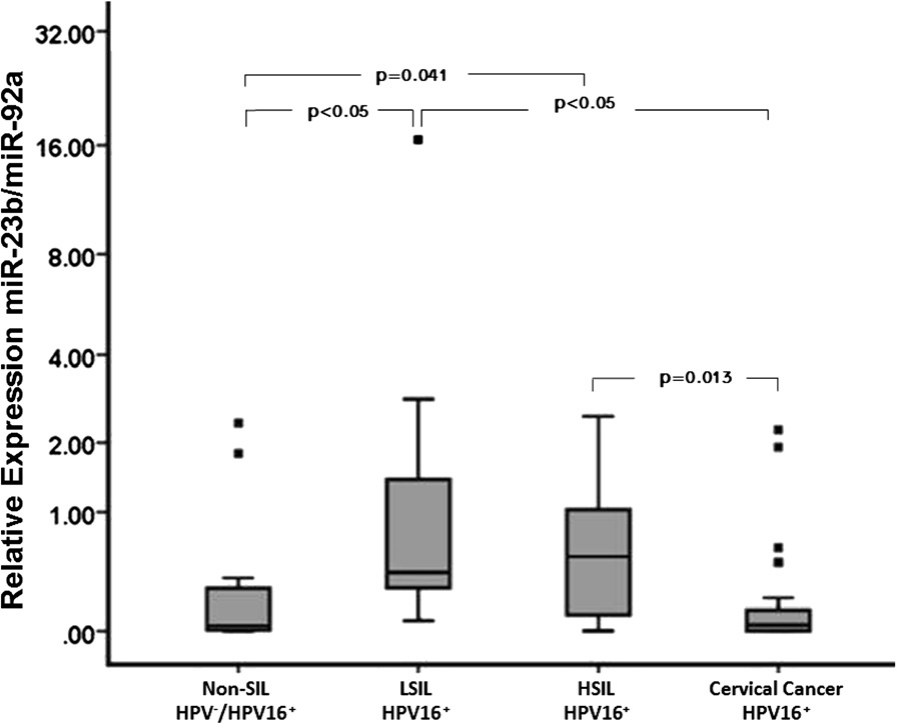
Expression of miR-23b in cervical scrapes from non-SILs and in tissues from patients diagnosed with LSIL, HSIL and cervical cancer. The expression of miR-23b was significantly lower in cervical cancer biopsies compared with LSIL and HSIL tissues. miR-92a was used for normalization of the data. *Statistically significant difference (p < 0.05)

The expression of miR-23b was significantly lower in non-SIL samples (p < 0.05) and in cervical cancer tissues (p < 0.05) than in tissues from patients diagnosed with LSIL and HSIL, (Fig. 4).

Risk analysis for the expression of miR-23b indicated that the decrease in the expression levels of miR-23b in precursor lesions with HPV16 increased the risk of developing cervical cancer by 36-fold (OR = 36; 95 % CI =6.7-192.6; p < 0.05) (Table 1).

**Table 1:** Risk analysis for the expression of miR-23b in cervical tissues

Expression of miR-23b	Clinical diagnosis		OR (95 % CI)	Value of *p*
	SILs (n = 26)	Cervical Cancer (n = 28)		
Low level	2 (7.7 %)	**21 (75 %)**	**36 (6.7-192.6)**	<0.05
High level	**24 (92.3 %)**	7 (25 %)	1.0*	

*SIL* LSIL + HSIL, *OR* Odds ratio

95 % confidence interval

*Reference value

The bold data indicate the frequency of the expression level found in premalignant lesions and cervical cancer.

## Discussion

Although cervical cancer is one of the most widely studied tumor models, the role played by epigenetic factors such as the expression of miRNAs and DNA methylation in tumorigenesis is not yet fully understood.

The most significant findings of this study were as follows: 1) the miR-23b promoter is methylated in cervical cancer cell lines; 2) the expression of miR-23b is low in cervical cancer cell lines; 3) the expression of miR-23b increases significantly in HeLa, SiHa, CaSki and C33A cells after treatment with 5′-Aza-CdR; 4) the expression of miR-23b is higher in LSIL than in HSIL and cervical cancer, that is, it decreases as the grade of the lesion increases; 5) in biopsies positive for HPV16 cervical cancer, the expression level of miR-23b is similar to that found in HeLa, SiHa, CaSki and C33A cell lines, and 6) the expression of uPa, c-Met and Zeb1, which are likely targets for miR-23b, is different among cervical cancer cell lines.

Our results on the methylation of miR-23b in cervical cancer cell lines are consistent with those found by Majid *et al.,* in prostate cancer cell lines and tumor tissues. In cervical cancer, it is likely that the expression of miR-23b is also regulated by dinucleotides methylation in the CpG islands located upstream of the TSS within of its promoter.

Corresponding to the methylation level of the miR-23b promoter, which is close to 100 %, the expression of miR-23b was found to be low in cervical cancer cell lines, suggesting that methylation is the mechanism of regulation of the expression of miR-23b in these cells. Interestingly, we found that in CaSki and HeLa cells, which have a greater number of integrated viral copies, the expression of miR-23b was decreased more than in the SiHa cells with fewer integrated copies. While in C33A cells, which are HPV-negative, the lowest expression of miR-23b was detected. The difference in the expression level of miR-23b in the cervical cancer cell lines can be explained by the functional characteristics of each cell line and by the different molecular events as follows: a) tissue origin of each cell line; b) expression of miR-23b transcription factors; and c) multiple mechanisms of regulation for genetic expression.

In cells untreated with 5′-Aza-CdR, the miR-23b expression was higher in SiHa cells than in HeLa, CaSki and C33A cells, which can be explained, at least in part, by the differential expression of transcription factors that modulate the expression of miR-23b. SiHa cells express functional p53 protein at higher levels than in HeLa, CaSki and C33A cells [24]. p53 is a transcription factor [24] that modulates the expression of various genes. Bisio *et al.* found that miR-23b contains a consensus sequence with low-affinity binding for p53 upstream of the TSS. The authors concluded that cis regulation by p53 partially modulates the expression of miR-23b [22]. In SiHa cells, Au Yeung *et al.* found that p53 expression correlates with miR-23b expression when the HPV16 E6 oncoprotein is silenced [25]. The higher miR-23b expression level in SiHa cells can be a consequence of the p53 expression level.

In C33A cells, p53 is expressed at normal levels but is not functional because it has a point mutation at codon 273 in an evolutionarily conserved domain. This mutation results in the substitution of an arginine with a cysteine [24]. It is likely that mutated p53 is unable to transactivate miR-23b, which would explain the low levels of this miRNA in HPV-negative cervical cancer cells. Other factors that may be influencing the expression of miR-23b and other microRNAs are as follows: the HPV type; the number of viral genome copies integrated into the cell genome [2, 26]; genetic polymorphisms or mutations in the promoter of the transcription factors that modulate the expression of miR-23b [27, 28]; defects in the molecular machinery responsible for the biogenesis of miR-23b [29, 30]; and the degree of methylation of miR-23b [7, 8].

We found that the expression of miR-23b is higher in LSILs than in HSILs and cervical cancer, that is, the expression of this miRNA decreased as the grade of the lesion increased. The decreased expression of miR-23b in biopsies from patients with cervical cancer positive for HPV-16 was similar to that found in HeLa, SiHa, CaSki and C33A cells. miR-23b overexpression in LSILs suggested that miR-23b in cervical carcinogenesis is a tumor suppressor.

The significantly lower miR-23b expression in biopsies of cervical cancer than in LSILs and HSILs, may be the result of gradual miR-23b promoter methylation and alterations of other gene expression regulatory mechanisms that are frequent in carcinogenesis. During tumorigenesis, cells undergoing epithelial-mesenchymal transition (EMT) are subject to metaplasia, which is characterized by the loss of E-cadherin (CDH1) expression [31, 32]. In hepatocellular carcinoma cell lines, decreased levels of miR-23b are correlated with the loss of expression of CDH1 [31, 32]. In endometrial carcinosarcoma, miR-23b inhibits the expression of mesenchymal markers [33]. Evidence indicates that CDH1 expression is downregulated by the snai1 and zeb1 transcription factors, which are molecular targets of miR-23b [31–33].

The low miR-23b expression in cervical scrapes can be explained by the origin of the sample, which determines the type and features of the cells present in the scrape. Cervical scrapes were obtained from the cell transformation zone (TZ). The transformation zone contains highly undifferentiated normal cells, such as metaplastic cells that generally lose cell-cell junctions due to the decreased expression of CDH1 [31, 32]. In endothelial cells, miR-23b expression is negatively associated with genes that regulate the cell cycle. During the cell cycle, phosphorylated pRb induces G1/S cell-cycle transition [34]. Low miR-23b expression is associated with the phosphorylation status of pRb and indirectly with increased E2F expression, thus promoting the proliferation of endothelial cells [35]. In metaplastic cervical cells, the decreased miR-23b expression may be correlated with continuous pRb activity and cell proliferation.

The similarity in miR-23b expression levels between cervical scrapes and biopsies of cervical cancer may be the result of the phenotypic similarity of squamocolumnar junction cells (also known as TZ) with cancer cells and HSIL cells. The subset of cuboidal cells in the squamocolumnar junction, which give rise to cancer, and the cancerous tissue maintain a common partial profile of gene expression [36, 37]. A high percentage of LSIL and HSIL disappear spontaneously [37] and is highly likely that in such cases the expression level of miR-23b is increased compared with Non-SIL. Thus, the expression level of miR-23b in SILs and cervical cancer agree with a tumor suppressor gene.

Evidence indicates that uPa, c-Met and Zeb1 are important promoters of tumor phenotype. uPA is a serine protease that modulates the turnover of the extracellular matrix and is related to metastatic tumor phenotypes [38]. c-Met is recognized as a factor that induces cell migration, and Zeb1 is recognized as a factor that promotes epithelial-mesenchymal transition [32]. In highly malignant cervical cancer tissue, the expression of uPA, the loss of expression of CDH1 and nuclear expression of snai1 and zeb1 are strongly associated with advanced stages of cervical cancer and lymph node metastasis [32, 39].

In this study, the mRNA expression levels of uPa, c-Met and Zeb1, which are likely targets of miR-23b, were compared in HeLa, SiHa, CaSki and C33A cells with and without exposure to 5′-Aza-CdR. The expression levels of *uPa* decreased in CaSki and C33A cells after treatment. These findings partially agreed with those reported by Au Yeung *et al.,* who demonstrated that the overexpression of miR-23b decreases the expression of uPa in SiHa and CaSki cells. The increase in the expression of miR-23b after treatment with 5′-Aza-CdR may not be sufficient to inhibit the expression of uPa in SiHa and HeLa cells. In contrast, uPa expression is also influenced by the level of expression and activity of its transcription factors [40] as well as by methylation of its promoter region [41]. In cervical cancer tissue, the HR-HPV infection and subsequent alteration of miRNA expression contribute to deregulate the expression of uPa [25]. However, there is still insufficient evidence to state that miR-23b directly regulates the expression of uPa in this tissue.

The expression level of *c-Met* was significant lowest in HeLa cells after treatment with 5′-Aza-CdR, which agreed with data reported by Salvi *et al.* [38], who proposed that miR-23b is a regulator of c-Met expression. Although *zeb1* is a direct target of miR-23b in bladder cancer [42], there are no current studies that support this theory in cervical cancer. We found that the expression levels of *Zeb1* decrease significantly in C33A and CaSki cells after being treated with 5′-Aza-CdR, suggesting that miR-23b is involved in regulating the expression of *Zeb1* in these cells. Interestingly, in SiHa cells, the hypomethylating treatment resulted in increased expression of *uPa, c-Met* and *Zeb1*, thereby suggesting that other molecular mechanisms affected by changes in the levels of DNA methylation independent of miR-23b are involved in regulating these genes in SiHa cells.

To explain the differential expression of uPa, c-Met and Zeb1 in cervical cancer cell lines, it should be noted that miRNAs repress or stimulate gene expression in response to specific cellular conditions, sequences and cofactors. The biological outcome of the miRNA-mRNA interaction is influenced by the following factors: the percentage of base pairing between the miRNA and target site; the number and relative position of target sites for the same miRNA; accessibility of the site; sequences flanking the target site; and the mRNA secondary structure, which may influence the hybridization sequences [43]. Moreover, Vasudevan and Steitz reported that the gene repression mediated by a miRNA may be reversible [44]. Thus, the expression of uPa, c-Met and Zeb1*,* which are likely targets of miR-23b, can be the result of specific features of each cell type, but it is also likely that some of these mRNAs were translated into protein at the time of RNA collection. More detailed studies are needed to determine if uPa, c-Met and Zeb1 are genes regulated by miR-23b in cervical cancer.

## Conclusions

In cervical cancer cell lines, miR-23b expression is regulated by its promoter methylation, and is likely the same process that occurs in cervical carcinogenesis. The decrease in miR-23b expression level is associated with cervical cancer, suggesting that this miRNA is a tumor suppressor in this cancer. The expression of uPa, c-Met and Zeb1 in cervical cancer cell lines is likely influenced by miR-23b, and the difference in the result of the miRNA-mRNA interaction is determined by factors related to the specific microenvironment of each cell type, with unique recognition sequences, cofactors and events that influence the biological outcome.

## Material and methods

### Culture of cervical cancer cell lines

The following cell lines were used: HeLa (50 copies of integrated HPV 18), SiHa (1–2 copies of integrated HPV16), CaSki (450 to 600 copies of integrated HPV16) and C33A (HPV-negative). The cells were cultured in Dulbecco′s modified Eagle medium (DMEM) supplemented with fetal bovine serum (10 %) and penicillin/streptomycin (1 %) (Invitrogen, Carlsbad, CA, USA). The cells were incubated at 37 °C in humidified atmosphere with 5 % CO_2_ [15, 45].

### Treatment of cell lines with 5′-Aza-2-deoxycytidine (5-Aza-CdR)

Cells were seeded in 6-well plates (25 × 10^3^ cells/well) and cultured for 72 h before treatment. The cells were treated with 10 μM 5-Aza-CdR dissolved in DMSO and added to fresh culture medium. The cultures were incubated at 37 °C in 5 % CO_2_ for 24 h. The treatment was then repeated, and the incubation was continued for an additional 24 h under the same conditions [46, 47]. The assay was performed in triplicate for each cell line. Untreated cells were included as a control [45].

### Patients and cell or cervical tissue samples

We studied 54 biopsies from women with cytopathological and histopathological diagnosis of squamous intraepithelial lesions (SILs) or cervical cancer with infection by HPV16. Of these biopsies, 19 were of low-grade squamous intraepithelial lesions (LSILs), 7 were of high-grade squamous intraepithelial lesions (HSIL) and 28 were of cervical cancer. The samples were obtained during routine screening for detection of premalignant lesions or cervical cancer at the State Cancer Institute “Dr. Arturo Beltran Ortega” in Acapulco, Guerrero. We included 18 cervical scrapings from women who had cervical cytologies without squamous intraepithelial lesions (non-SILs) and who were infected with or without HPV16. These women attended the Immunohistochemistry and Cytopathology Laboratory of the Autonomous University of Guerrero (Chilpancingo, Guerrero, Mexico) for timely detection of cervical cancer.

Before sampling, all women signed an informed consent to participate in the study.

### Extraction and purification of nucleic acids

Extraction of total RNA and DNA from the cell lines before and after treatment with 5-Aza-CdR as well as from cervical samples was performed with TRIzol reagent according to the manufacturer’s instructions. The integrity of both nucleic acids was determined by electrophoretic shift in a 1 % agarose gel [48]. The DNA was stored at −20 °C, and the RNA was stored at −70 °C.

### Detection and typing of HPV16

Detection and typing of HPV was performed using the INNO-LiPA genotyping extra kit (Innogenetics, Barcelona, Spain) according to the manufacturer’s instructions.

### Methylation analysis of miR-23b by RT-PCR

The methylation status of the promoter region of miR-23b was determined using the Human cancer miRNA EpiTect Methyl II Signature PCR Array® (QIAGEN Sciences, Maryland, USA) following the manufacturer’s instructions. Briefly, this assay was based on the digestion of methylated and unmethylated DNA using methylation-sensitive and methylation-dependent restriction enzymes. The DNA that remained after digestion was added to the matrix. The ABI 7500 system for real-time PCR was used to read the plates. The relative amount of methylated and unmethylated DNA was calculated using the standard ∆Ct method, normalizing the amount of DNA in each digestion against the total amount of input DNA in a null digestion using an Excel spreadsheet provided by the manufacturer.

### Quantitative analysis of miR-23b expression by RT-PCR

The expression of miR-23b was determined using the ABI 7500 system for real-time PCR (Applied Biosystems, Foster City, CA). Reverse transcription was performed using 10 ng of total RNA. The expression of miR-23b (000400, AUCACAUUGCCAGGGAUUACC) was measured using TaqMan microRNA assays following the manufacturer’s instructions (Applied Biosystems). The expression of miR-92a (000431, UAUUGCAUUGUCCCGGCCUGU) was used as an internal reference for the expression of miR-23b. The relative expression of both miRNAs was analyzed by the comparative Ct method.

### Quantitative polymerase chain reaction (qPCR) assays of upa, c-met and zeb1

The qPCR assays were performed according to the manufacturer’s instructions with a One-Step qRT-PCR kit (KAPA SYBR® FAST, Boston, Massachusetts, USA). Using qPCR assays, we assessed mRNA expression levels of the following genes: uPa (5′-GTCGTGAGCGACTCCAAAGGCA-3′ and 5′- GGGCAGTTGCACCAGTGAATGT-3′), c-Met (5′-TATTTCCCAGATCATCCATTGCA-3′ and 5′- AATGTAGGACTGGTCCGTCAAAA-3′) and Zeb1 (5′- GCACCTGAAGAGGACCAGAG-3′ and 5′- TGCATCTGGTGTTCCATTTT-3′). We used GAPDH (5′ GGTGAAGGTCGGTGTGAACG-3′ and 5′ CTCGCTCCTGGAAGATGGTG-3′) as the internal reference. The thermal cycling profile was as follows: cDNA synthesis step was performed using 200 ng of total RNA at 42 °C for 5 min, followed by an inactivation RT step at 95 °C for 5 min, and 40 cycles of a denaturation step at 95 °C for 3 s, an annealing/extension step at 60 °C for 30 s, and a dissociation step according to the instrument guidelines. The qPCR assay was independently repeated three times using the StepOne system for real-time PCR (Applied Biosystems, Foster City, CA). The relative expression of uPa, c-Met and Zeb1 was analyzed by the comparative Ct method.

### Data analysis

Using the STATA statistical package (version 9.2), we determined the frequency of methylation of the promoter region of miR-23b, and the same statistical package was used to determine the expression changes of miR-23b, uPa, c-Met and Zeb1 between cells treated with 5′-Aza-CdR and untreated cells. Fisher’s exact test was used to compare the differences between the two conditions. The median and interquartile range of the miR-23b expression was determined, and the difference between groups was calculated by the Mann-Whitney’s test. The association between the expression of miR-23b and the presence of cervical cancer was determined by logistic regression with a confidence level of 95 %. A value of p < 0.05 was considered statistically significant.

## Abbreviations

HR-HPV: High-Risk Human Papillomavirus
microRNA: miRNA
uPA; *upa*: Urokinase-type Plasminogen Activator
DNMT1: DNA methyltransferase 1
5′-Aza-CdR: 5′Aza-2-deoxycytidine
Non-SIL: Without squamous intraepithelial lesion
SIL: Squamous intraepithelial lesion
LSIL: Low-grade squamous intraepithelial
HSIL: High-grade squamous intraepithelial lesion
CDH1: E-cadherin
EMT: Epithelial-Mesenchymal Transition
Snai1: Zinc finger protein SNAI1
ZEB1: Zinc finger E-box-binding homeobox 1 and 2
DMSO: Dimethylsulfoxide
DMEM: Dulbecco’s modified eagle médium
